# Decreased plasticity of coreceptor use by CD4-independent SIV Envs that emerge *in vivo*

**DOI:** 10.1186/1742-4690-10-133

**Published:** 2013-11-12

**Authors:** Nicholas Francella, Sarah TC Elliott, Yanjie Yi, Sarah E Gwyn, Alexandra M Ortiz, Bing Li, Guido Silvestri, Mirko Paiardini, Cynthia A Derdeyn, Ronald G Collman

**Affiliations:** 1 Department of Medicine, University of Pennsylvania Perelman School of Medicine, 522 Johnson Pavilion, 36th & Hamilton Walk, Philadelphia, PA, USA; 2 Department of Pathology and Laboratory Medicine and the Yerkes National Primate Research Center, Emory University, Atlanta, GA, USA

## Abstract

**Background:**

HIV and SIV generally require CD4 binding prior to coreceptor engagement, but Env can acquire the ability to use CCR5 independently of CD4 under various circumstances. The ability to use CCR5 coupled with low-to-absent CD4 levels is associated with enhanced macrophage infection and increased neutralization sensitivity, but the additional features of these Envs that may affect cell targeting is not known.

**Results:**

Here we report that CD4-independent SIV variants that emerged *in vivo* in a CD4+ T cell-depleted rhesus macaque model display markedly decreased plasticity of co-receptor use. While CD4-dependent Envs can use low levels of macaque CCR5 for efficient entry, CD4-independent variants required high levels of CCR5 even in the presence of CD4. CD4-independent Envs were also more sensitive to the CCR5 antagonist Maraviroc. CD4-dependent variants mediated efficient entry using human CCR5, whereas CD4-independent variants had impaired use of human CCR5. Similarly, CD4-independent Envs used the alternative coreceptors GPR15 and CXCR6 less efficiently than CD4-dependent variants. Env amino acids D470N and E84K that confer the CD4-independent phenotype also regulated entry through low CCR5 levels and GPR15, indicating a common structural basis. Treatment of CD4-dependent Envs with soluble CD4 enhanced entry through CCR5 but reduced entry through GPR15, suggesting that induction of CD4-induced conformational changes by non-cell surface-associated CD4 impairs use of this alternative co-receptor.

**Conclusions:**

CD4 independence is associated with more restricted coreceptor interactions. While the ability to enter target cells through CCR5 independently of CD4 may enable infection of CD4 low-to-negative cells such as macrophages, this phenotype may conversely reduce the potential range of targets such as cells expressing low levels of CCR5, conformational variants of CCR5, or possibly even alternative coreceptors.

## Background

During HIV and SIV entry, gp120 engagement of CD4 is normally required to initiate the conformational changes in Env that form and expose a coreceptor binding site, which then allows CCR5 engagement and subsequent entry steps to occur [1,2]. Although extremely rare in natural infection *in vivo*, several pathways have been described by which HIV and SIV can adapt to use CCR5 in the presence of little-to-no CD4 [3-9]. The factors that serve to restrain CD4 independence during normal infection, and their relationship to coreceptor use and entry, have important implications for cell targeting and tropism *in vivo*.

We recently described a unique SIV model in which such CD4-independent Env variants emerged and dominated in the plasma of multiple rhesus macaques experimentally depleted of peripheral (but not mucosal) CD4+ T cells prior to infection [9,10]. These animals displayed widespread infection of tissue macrophages, which express very low levels of CD4 compared to CD4+ T cells, indicating a mechanism by which virus could expand its target cell range in this setting of limited CD4+ T cell targets. The CD4-independent Env variants that arose in CD4+ T cell depleted macaques were highly sensitive to antibody neutralization, a characteristic that is common among previously-described CD4-independent HIV and SIV [5-8]. Unlike the CD4-independent Envs that were constitutively sensitive to neutralization by normal SIV + plasma and monoclonal antibodies, CD4-dependent control Envs became sensitive only if first incubated with soluble CD4 (sCD4). CD4+ T cell-depleted animals failed to produce this CD4-inducible neutralization activity, however, enabling emergence of CD4-independent variants in these animals. These findings, along with other studies [5-8], suggest that antibodies shape the cellular tropism of the virus *in vivo* by preventing the emergence of CD4-independent variants during typical infection. Both CD4 independence and neutralization sensitivity were conferred by D470N/E84K mutations in Env that arose in these animals [9], indicating that these are linked phenotypes resulting from a common structural basis.

In this study, we wished to determine whether there were additional biological features of CD4-independent Envs that arise in this model that, in addition to sensitivity to antibody neutralization, might affect cell entry and targeting, and thus impact emergence of such variants. It has previously been reported that some HIV isolates that can enter cells using low levels of CD4 may be limited in their capacity to efficiently engage low levels of CCR5 [11,12]. Since most CD4+ T cells express relatively low levels of CCR5 [13-17], this property could impact cell targeting by restricting infection *in vivo* to the limited subset of cells expressing high levels of CCR5. In addition, CCR5 is expressed on primary cells in a variety of conformational forms, suggesting that efficient infection may require some degree of plasticity in Env function that allows it to interact with such conformational variants [18-20]. Thus, seeking to understand whether more restricted coreceptor use might be a factor, in addition to immune pressure, that could limit the emergence of CD4-independent Env *in vivo*, we tested our panel of plasma-derived CD4-independent Envs for their ability mediate entry using low-level CCR5, using human CCR5, and using the alternative coreceptors GPR15 and CXCR6. Low-level CCR5 clearly reflects a biologically important feature of primary target cells in vivo [13-15], while use of the human CCR5 molecule and of alternative coreceptors [21,22], may reflect a degree of “plasticity” by which Env can function using related but structurally distinct molecules.

Here we report that CD4-independent SIV Env variants that arise *in vivo* are impaired in their ability to use low levels of rhesus macaque CCR5 for entry even in the presence of CD4. Furthermore, they are significantly less efficient in use of human CCR5, and rhesus macaque GPR15 and CXCR6, than are CD4-dependent control Envs. Loss of coreceptor plasticity was conferred by the same molecular determinants that regulate CD4-independence, indicating a shared structural basis. These findings suggest that CD4-independent variants may have an expanded capacity for infection of CD4-low targets, but may have an otherwise narrower range of potential cellular targets *in vivo* due to more limited ability to infect CCR5-low cells and/or cells expressing CCR5 conformational variants. Thus, in addition to neutralization sensitivity, a more restricted coreceptor utilization capacity may also serve to limit the emergence of CD4-independent variants during normal infection.

## Results

### CD4-independent SIV Envs have reduced capacity compared to CD4-dependent Env to use human CCR5

We previously described a model for *in vivo* emergence of CD4-independent SIV in which rhesus macaques were experimentally depleted of CD4+ T-cells before SIVmac251 infection [9,10]. Envs isolated from the plasma of CD4+ T cell-depleted animals at day 42 (d42) post infection were capable of mediating infection using CCR5 independently of CD4, while Envs from CD4+ T cell-depleted macaques early after infection and control macaques both early and late after infection were strictly CD4-dependent [9].

**Figure 1:**
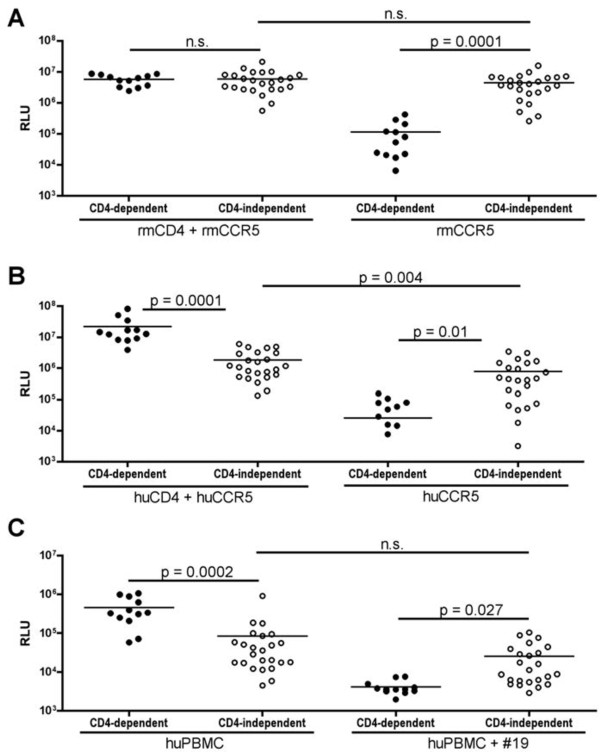
**Lower efficiency of human CCR5 use by CD4-independent compared to CD4-dependent SIV Envs.** Pseudotyped viruses carrying Envs from d42 CD4+ T cell depleted macaques (CD4-independent Envs; open circles) or control macaques (CD4-dependent Envs; closed circles) were used infect 293 T cells transfected with receptor molecules **(A,B)** or primary human PBMCs **(C)**. **(A)** 293 T cells were transfected with rhesus macaque (RM) CCR5 with or without rhesus CD4 and infected with pseudotype viruses. Entry was assessed based on luciferase production 72 hours after infection and expressed as relative light units (RLU). **(B)** SIV Env-pseudotyped viruses were used to infect 293 T cells transfected with human CCR5 with or without human CD4. **(C)** Envs were assessed for their ability to mediate viral entry into PHA/IL2-stimulated human PMBCs that were pre-treated with or without a CD4 blocking antibody (mAb #19). Unpaired T-tests were used to compare entry between the 2 sets of Envs in a given target cell type, while paired T-tests were used to compare entry of Envs in the presence versus absence of CD4.

To understand the features of CD4-independent Envs, we focused on 23 CD4-independent Envs from d42 plasma of four CD4+ T cell-depleted animals and 12 CD4-dependent Envs from d42 plasma of two control animals. As shown in Figure 1A, the CD4-independent Envs mediated equivalent levels of pseudotype virion entry into 293 T cells transfected with rhesus macaque CCR5 (rmCCR5) with and without rmCD4, whereas the control Envs were CD4-dependent. In addition, CD4-dependent and CD4-independent Env variants had equivalent levels of entry overall into cells expressing both rmCD4 and rmCCR5. Thus, the CD4-independent Envs are as efficient as CD4-dependent Envs at mediating entry using rmCCR5 in this transfection system, and are essentially indifferent to the presence or absence of CD4 when entering through rmCCR5.

In contrast to entry using RM receptors, the CD4-independent Envs were significantly less efficient than CD4-dependent Envs in entering cells expressing human CCR5 and CD4 (Figure 1B). The lower overall entry using human receptors was due to less efficient use of human CCR5, as entry in the presence of rmCCR5/huCD4 was as efficient as entry in the presence of rmCCR5/rmCD4, and entry using huCCR5/rmCD4 was similar to huCCR5/huCD4 (data not shown). The CD4-independent Envs were CD4-independent on human CCR5, although entry was lower in cells expressing huCCR5 alone compared to huCCR5/huCD4. Thus, the CD4-independent Envs are attenuated in their use of huCCR5 compared to CD4-dependent Envs.

We further examined whether the CD4-independent phenotype we observed during infection of cells over-expressing CCR5 also applied to primary cells, and whether the CD4-independent Envs were less efficient than CD4-dependent Envs in huCCR5 use in the context of primary cells (Figure 1C). Primary human PBMCs were treated with or without the CD4 monoclonal antibody #19, which blocks CD4-dependent HIV and SIV entry [23]. As expected, entry into PBMCs by CD4-dependent Envs was reduced to background levels by mAb #19, whereas CD4-independent variants were largely resistant to entry blockade by #19, confirming that the CD4-independent phenotype extends to primary cells as well. Overall entry by CD4 independent viruses (in the absence of mAb #19) was attenuated compared to CD4-dependent Envs, which stands in contrast to the equivalent entry into rmCCR5/rmCD4 transfected cells for the CD4-independent and –dependent viruses (Figure 1A), but is similar to lower entry for the CD4-independent viruses into huCCR5/huCD4 transfected cells (Figure 1B). Thus, CD4-independent variants are also less efficient than control Envs at entry into human CCR5/CD4-expressing primary cells. The attenuated entry by CD4-independent viruses likely reflects less efficient use of human CCR5, but since primary cells express CCR5 at lower levels than transfected cells, could also reflect inefficient use of lower levels of coreceptor compared with CD4-dependent Envs.

### CD4-independent SIV Envs use low levels of rmCCR5 less efficiently than CD4-dependent Envs

**Figure 2:**
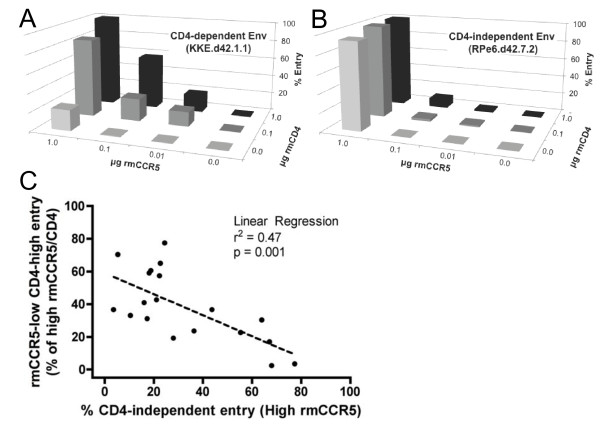
**CD4-independence is associated with reduced ability to use low levels of rmCCR5.** SIV Env-pseudotyped viruses were tested for their ability to infect 293 T cells transfected with different amounts of rmCD4 and rmCCR5. **(A,B)** 3D plots showing entry by representative CD4-dependent **(A)** and CD4-independent **(B)** Envs. The horizontal (X) axis indicates the amount of transfected CCR5 decreasing from left to right, the depth (Z) axis indicates the amount of transfected CD4 decreasing from back to front, and the vertical (Y) axis indicates the level entry with 100% set at entry in the presence of maximal rmCD4 and rmCCR5. **(C)** The panel of Envs were tested as in **A** and **B**, with the X axis indicating CD4-independent entry (no CD4, maximal rmCCR5) and Y axis indicating entry via low CCR5 (maximal rmCD4 but only 10% of maximal rmCCR5). Each Env is indicated as a single point on the plot. Linear regression analysis was used to assess the correlation between CD4-independent entry and low-CCR5 entry.

To address the impact of CCR5 expression levels, we tested the ability of CD4-independent SIV Env variants to mediate entry into 293 T cells transfected with various amounts of rmCD4 and rmCCR5 (Figure 2). Flow cytometry analysis showed that transfection of 0.0, 0.01, 0.1, or 1.0 μg rmCCR5 plasmid resulted in mean fluorescence intensity (MFI) levels of approximately 5, 6, 10, and 29, respectively, while transfection with 0.0, 0.1, or 1.0 μg rmCD4 plasmid resulted in MFI levels of 4, 9, and 15, respectively (data not shown), confirming that varying plasmid transfection results in correspondingly varying levels of receptor surface expression.

As expected, CD4-dependent control Envs exhibited high infectivity at high levels of CCR5, which gradually dropped at lower levels of CCR5 (Figure 2A). In contrast, CD4-independent Envs exhibited a steep decline in infectivity even at slightly reduced levels of CCR5 (Figure 2B). We then analyzed a larger panel of Envs with this assay, and determined the relationship between CD4-independence and the ability to use low levels of CCR5 (Figure 2C). This analysis revealed a strong inverse correlation between entry into cells expressing low levels of rmCCR5 (in the presence of CD4) and entry into cells expressing high levels of rmCCR5 without CD4 (p = 0.001). These results indicated that among SIV Env variants that arise *in vivo* in infected macaques, CD4-independence is strongly associated with inefficient use of low levels of CCR5, even in the presence of CD4.

### CD4-independent Envs show increased sensitive to the CCR5 inhibitor Maraviroc

Sensitivity to small molecule inhibitors of CCR5 is often used as a surrogate for efficiency of Env-CCR5 interactions, with less efficient interactions associated with greater sensitivity [14]. We therefore tested whether the CD4-independent Envs would differ from CD4-dependent Envs in sensitivity to the CCR5 inhibitor maraviroc. Target 293 T cells were transfected with rmCD4 and rmCCR5, pre-treated with or without varying concentrations of maraviroc, and infected with Env-pseudotyped viruses.

**Figure 3:**
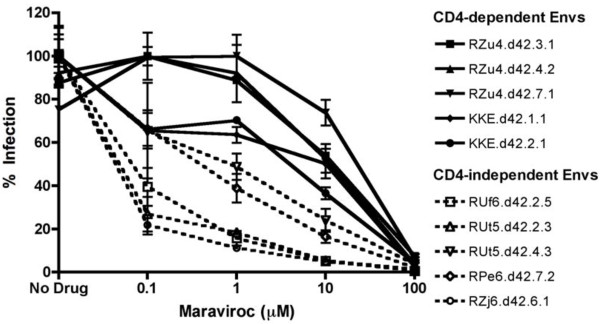
**Maraviroc sensitivity of CD4-independent Envs.** Pseudotyped viruses carrying CD4-independent (dotted lines) or control (solid lines) SIV Envs were tested for sensitivity to inhibition by maraviroc. Target cells were 293 T cells transfected with high levels of rmCD4 and rmCCR5 (1 μg each) and treated with various concentrations of maraviroc for 1 hour prior to and during infection.

As shown in Figure 3, maraviroc inhibited entry of CD4-dependent and CD4-independent Envs in a dose-dependent manner, as expected. However, there was a striking difference, with all CD4-independent Envs tested showing markedly greater sensitivity than all of the CD4-dependent Envs (p < 0.01 by Wilcoxon rank-sum test). Thus, like the less efficient use of low level CCR5, CD4-independent Envs are impaired in their ability to use CCR5 in the presence of a small molecule antagonist.

### Decreased efficiency of GPR15 and CXCR6 use by CD4-independent Envs

**Figure 4:**
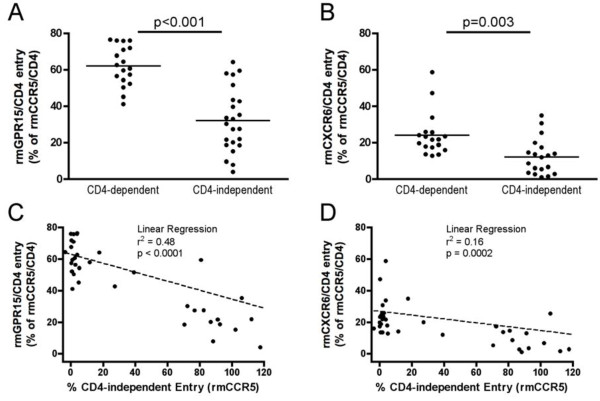
**Reduced entry using GPR15 and CXCR6 by CD4-independent Envs.** Env-pseudotyped viruses were tested for their ability to infect cells transfected with rmCD4 and either rmGPR15 or rmCXCR6. **(A,B)** Entry into 293 T cells transfected with rmCD4 and either rmGPR15 or rmCXCR6 was measured for each Env, and is indicated as a percentage of entry by that Env using rmCCR5 and rmCD4. **(C,D)** Envs from **(A)** or **(B)** were plotted showing entry in cells transfected rmCCR5 without CD4 on the X axis, and entry into cells transfected with rmCD4 and either rmGPR15 **(C)** or rmCXCR6 **(D)** on the Y axis. Each Env is indicated as a single point on the plot, and linear regression analysis was performed to assess the relationship between CD4-independent entry and GPR15 or CXCR6 use.

In addition to CCR5, several other G protein-coupled receptors can be used as entry co-receptors by SIV *in vitro*, reflecting a substantial degree of plasticity in the molecules with which these Envs can interact. To determine whether the efficiency of alternative coreceptor use differed between CD4-dependent and independent Envs, we assessed their ability to enter cells transfected with either the orphan receptor GPR15 or the chemokine receptor CXCR6, in conjunction with CD4 (Figure 4).

CD4-dependent control Envs mediated efficient entry into rmGPR15/rmCD4-expressing cells, reaching a mean of 61% (range 40-80%) of entry compared to rmCCR5/rmCD4, which confirms that GPR15 is used by these SIVmac251 variants. However, CD4-independent Envs were significantly impaired in their use of GPR15 compared to control Envs (mean of 30%; range 2-60%). Regression analysis showed a highly significant inverse correlation between the ability of Env to use CCR5 in the absence of CD4 and the ability to use GPR15 in the presence of CD4 (p < 0.0001; Figure 4C), indicating that CD4 independence is associated with decreased efficiency of GPR15 use.

Consistent with previous studies [21], most SIVmac Envs used rmCXCR6 poorly compared to CCR5 and GPR15 (Figure 4C). Entry mediated by control Envs into rmCXCR6/rmCD4-expressing cells reached a mean of 23% (range 10-60%) of entry compared to rmCCR5/rmCD4. Similar to GPR15, CD4-independent Envs were significantly impaired in their use of CXCR6 (mean of 10%; range 0-35%). Regression analysis showed a highly significant inverse correlation between the ability of Env to use CCR5 in the absence of CD4 and the ability to use CXCR6 in the presence of CD4 (p = 0.0002; Figure 4D), indicating that CD4 independence is also associated with decreased efficiency of CXCR6 use.

### D470N and E84K regulate efficiency of low-CCR5 use and GPR15 use

Since the CD4-independent variants were all derived from plasma of macaques experimentally depleted of CD4+ T cells prior to infection, the decreased efficiency of alternative coreceptor and low-CCR5 use among these variants could conceivably have resulted from selective pressures on these Envs *in vivo* unrelated to their CD4-independent phenotype. Therefore, in order to ask whether CD4 independence and decreased low-CCR5 and alternative coreceptor use were inextricably linked, we asked whether the molecular determinants of CD4-independence also regulated these characteristics. We recently identified two amino acids that emerged in the CD4+ T cell-depleted macaque Envs that conferred CD4 independence when introduced into CD4-dependent Envs (D470N, E84K), and which abrogated CD4 independence when reverted in the CD4-independent Envs [9]. E84K and D470N led to charge changes in and around the predicted CD4-binding site in gp120, with D470N introducing a potential N-linked glycosylation site that was important for CD4-independent entry. As individual mutations E84K and D470N each contributed to the CD4-independent phenotype but attenuated overall entry, whereas together they conferred robust entry in the presence of rmCCR5 both with and without CD4 [9].

**Figure 5:**
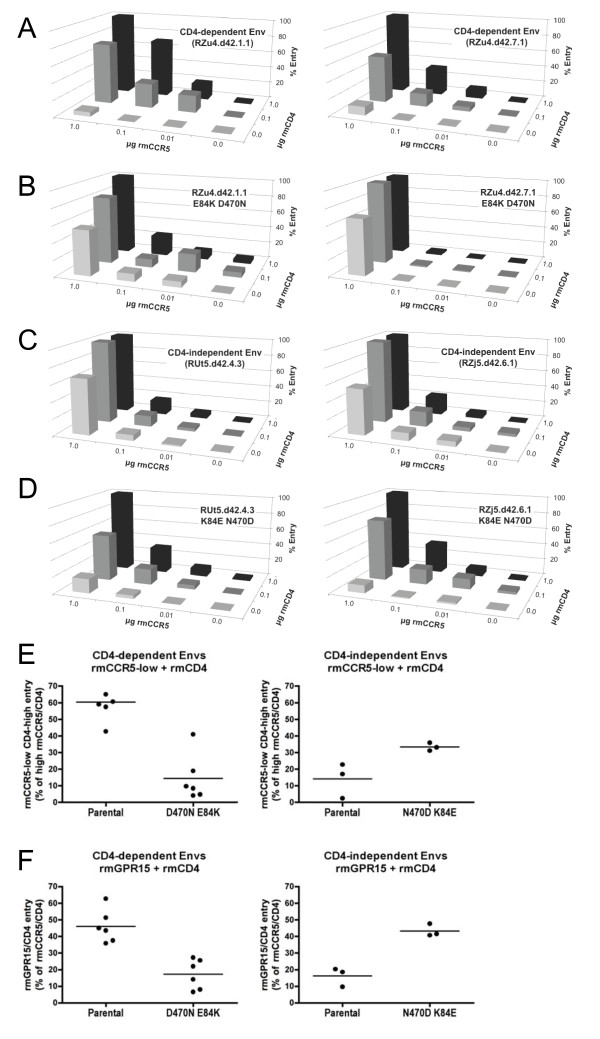
**Env D470N and E84K determine efficiency of CCR5 and GPR15 use.** Mutations that emerged *in vivo* which conferred CD4 independence (D470N and E84K) were introduced into CD4-dependent Envs, and mutations that abrogate CD4 independence (N470D and K84E) were introduced into CD4-independent Envs. **(A-D)** Two representative parental CD4-dependent Envs **(A)** and their respective mutated forms **(B)** and two representative parental CD-independent Envs **(C)** and their respective mutated forms **(D)**, were assessed for their ability to mediate entry into cells transfected with varying amounts of rmCCR5 and rmCD4, as described in Figure 2. **(E)** Use of low rmCCR5 levels by all parental and mutant Envs (10% maximal CCR5 in the presence of maximal CD4), with each Env indicated as a dot and entry in the presence of maximal CCR5/CD4 set at 100%. **(F)** Use of GPR15 by parental and mutated Envs were tested based on entry into cells transfected with rmGPR15 and rmCD4, with each Env indicated as a dot and expressed as a percentage of entry in the presence of rmCCR5/rmCD4.

When D470N and E84K were introduced into control Envs, they conferred CD4 independence but markedly reduced the ability of virons to enter into cells expressing low levels of rmCCR5 even in the presence of CD4 (Figure 5A,B,E). Conversely, when N470D and K84E were introduced into CD4-independent Envs, they reduced the ability to utilize rmCCR5 independently of CD4, but enhanced the ability to utilize low levels of rmCCR5 in the presence of CD4 (Figure 5C,D,E). K84E/N470D did not completely restore use of low CCR5 to the level of CD4-dependent Env (Figure 5E), suggesting that additional determinants may also contribute to efficiency of CCR5 use. Finally, introduction of D470N and E84K into control Envs reduced use of rmGPR15, while N470D and K84E enhanced rmGPR15 use (Figure 5F). Thus, the molecular determinants that regulate CD4-independent use of CCR5 are also the molecular determinants of co-receptor use plasticity, indicating that they are linked properties of these Envs, rather than independent but co-selected phenotypes of virus variants that emerged in these animals.

### Conformational changes induced by soluble CD4 inhibit GPR15 use

**Figure 6:**
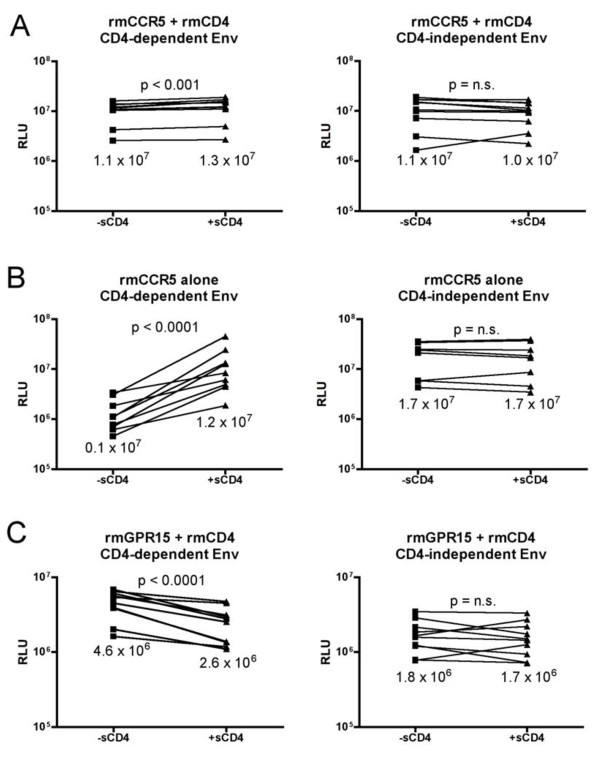
**Reduced use of GPR15 by SIV Envs pre-triggered with sCD4.** Pseudotype viruses carrying CD4-dependent or CD4-independent Envs were incubated with or without sCD4 (50 ug/ml for 1 hour) and assessed for their ability to infect cells 293 T cells expressing rmCCR5 and rmCD4 **(A)**, rmCCR5 without CD4 **(B)**, or rmGPR15 and rmCD4 **(C)**. Each Env is indicated as a box (without sCD4) and a triangle (with sCD4), and the two conditions for each individual Env are connected by a line. Paired T-tests were used to compare entry with and without sCD4 incubation.

Envs capable of using CCR5 in the absence of CD4 are thought to constitutively exist in a CD4-triggered conformation. Previous studies have shown that soluble CD4 (sCD4) does not efficiently inhibit SIV, and indeed is capable of triggering conformations in SIV Env that enable entry using CCR5 in the absence of CD4 [24,25]. To determine if sCD4 triggering has an effect on use of GPR15 similar to the CD4-independent phenotype, we tested the impact of sCD4 on the ability of CD4-dependent control Envs to mediate entry using rmGPR15/CD4. As shown in Figure 6, sCD4 pre-triggering enabled entry by CD4-dependent control Envs into cells expressing rmCCR5 in the absence of CD4, and slightly enhanced entry into cells expressing rmCCR5/CD4. In contrast, sCD4 treatment resulted in a significant (~50%) reduction in entry using rmGPR15/CD4 (Figure 6C), which is similar to the relative deficiency in GPR15 use by CD4-independent compared with control Envs (Figure 4A). Treatment of CD4-independent Envs with sCD4 had no impact on their ability to mediate entry using rmCCR5, with or without CD4, nor on their relative lack of ability to use rmGPR15/CD4. Thus, even though cell surface CD4 engagement is required for GPR15-mediated entry, CD4-induced conformational changes in Env that occur independent of cell surface CD4 inhibit the use of GPR15, concordant with the reduced ability of CD4-independent Envs to use GPR15 compared with CD4-dependent Envs. Notably, sCD4 did not inhibit entry mediated by control Envs in the presence of rmCCR5/CD4, suggesting that its inhibition of entry in the presence of rmGPR15/CD4 was not due to effects on cellular tethering, gp120 shedding, or similar mechanisms. Thus, sCD4 inhibition of entry using GPR15 is likely due to effects on gp120 interactions with the coreceptor.

## Discussion

We focused here on a panel of SIV Envs derived from the plasma of rhesus macaques that had been experimentally depleted of CD4+ T cells prior to infection, in which CD4-independent virus variants arose in multiple animals in association with widespread macrophage infection. The ability to use CCR5 in conjunction with low-to-absent CD4 is a common feature of viruses that efficiently infect macrophages, which express extremely low levels of CD4. However, while entry by these viruses using high levels of rmCCR5 in transfected cells is very efficient with or without CD4, the Envs display decreased efficiency of entry in four areas: ability to use low levels of CCR5; ability to use the human homologue of CCR5; sensitivity to a CCR5 entry inhibitor; and use of the alternative coreceptors GPR15 and CXCR6. Because these features are all tightly linked, we suggest that they reflect a common property of overall decreased “plasticity” of Env-coreceptor interactions, as a consequence of having acquired the ability to use CCR5 without CD4.

While multiple reports have described CD4-independent SIV and HIV generated in various model systems, strict CD4 dependence is a general rule for viruses *in vivo* during normal infection. Understanding the consequences of CD4 independence in settings where it is acquired, therefore, provides insight into critical factors that maintain CD4 dependence in normal infection. We previously reported that the CD4-independent variants in these animals are highly sensitive to neutralization by SIV + plasma and several monoclonal antibodies, similar to other CD4-independent variants described [5-9], whereas control Envs are resistant to neutralization unless pre-triggered by sCD4. Since the CD4+ T cell depleted animals in which these variants arose failed to produce such CD4-inducible neutralizing activity, one clear factor that appears to contribute to maintaining CD4 dependence is avoidance of plasma neutralization. However, our findings here that CD4-independent Envs exhibit more restricted coreceptor use suggest that additional factors may also impact function of CD4-independent Envs and regulate their relative fitness *in vivo*.

We found that CD4 independence is linked to defective use of low levels of CCR5 both phenotypically (Figure 2C) and structurally (Figure 5A-E). Decreased ability to use low levels of available CCR5 may also underlie CD4-independent Envs increased sensitivity to maraviroc (Figure 3 and [14]). In contrast to the CD4+ T cell depleted model in which these viruses arose, where a shift towards macrophage targets occurred, CD4+ memory T cells are the principal targets of HIV and SIV in normal infection. In general, CCR5 is expressed on CD4+ T cells at relatively low levels, in contrast to much higher expression on macrophages [15,16,26]. Indeed, low CCR5 expression is considered to be a principal factor limiting entry into CD4+ T cells [17], but not into macrophages, whereas low CD4 expression limits macrophage infection [27]. While the relationship between CCR5 density on primary macaque macrophages and lymphocytes compared with transfected 293 T cells has not been specifically defined, our data suggests that a loss of CCR5 efficiency accompanies CD4 independence among these Envs, which likely affects low-CCR5 but not high-CCR5 targets. Thus, while CD4 independence may enable the virus to infect an expanded range of CD4-low cell types, particularly macrophages, a consequence of this feature may be a restricted capacity to infect primary CD4+ T cells expressing low or moderate levels of CCR5.

CD4 independence of these Envs was also linked to less efficient use of human CCR5, despite equivalent entry in the presence of high levels of transfected rmCCR5 (Figure 1). In addition to overall lower levels of expression, CCR5 on primary CD4+ T cells exists in a range of structural conformations, which is reflected in conformational antibody and/or Env binding characteristics [16,17,19]. It has been previously suggested that the ability to engage alternative conformations of CCR5, or even the capacity to engage ligand-bound receptor, enables Env to more efficiently use the heterogeneous conformations that are expressed on CD4+ T cells [13-15,18-20]. CCR5 of human and macaque origin share 97.7% amino acid identity [28], and Env use of the two homologues is generally highly concordant. However, our observation that CD4-independent Envs are relatively impaired in use of huCCR5 compared with control Envs suggests that they are more sensitive to subtle structural variations in the molecule. We speculate that the greater use of huCCR5 that we observed in CD4-dependent Envs compared to CD4-independent Envs may reflect a greater ability to tolerate structural variations, which might allow these Envs to effectively engage CCR5 on a broader range of CD4+ T cells. Thus, in addition to restricted use of low levels of CCR5, reduced plasticity of coreceptor use may also result in a limited tolerance for structural variation, and thus a more limited range of CD4+ T cell targets for CD4-independent viruses.

The CD4-independent Envs in our study also displayed impaired entry into cells expressing rmGPR15 and rmCXCR6 compared to control CD4-dependent Envs, even in the presence of CD4. SIV Envs typically use a range of alternative coreceptors *in vitro*. However, while alternative entry pathways are used *in vivo* in nonpathogenic infection of sooty mangabey and red-capped mangabey natural hosts [29,30], it is unclear whether any of these pathways, including GPR15 and CXCR6, play a role in SIVmac infection of rhesus macaques [21,22], and there appears to be no consequence *in vivo* to loss of GPR15 utilization [22]. Thus, while we cannot completely exclude the possibility that less efficient GPR15 and CXCR6 use by CD4-independent variants may limit infection of GPR15+ or CXCR6+ cells *in vivo*, we think it is more likely that this feature reflects simply another aspect of the overall more coreceptor-constrained, less plastic function of the CD4-independent Envs.

Our observation that the ability of SIV to enter independently of CD4 correlates inversely with efficiency of CCR5 use differs from some previous studies that examined variants with the capacity to use exceedingly low levels of CD4 for entry, which were in some cases also better able to utilize low levels of CCR5 [31-34]. In those studies, the ability to scavenge very low levels of CD4 on the cell surface was thought to result from increased affinity for CD4, or more stable interactions, thus more efficiently triggering the CD4-induced conformational changes that enable coreceptor engagement [35]. In contrast to those studies, our CD4-independent variants were not sensitive to inhibition by sCD4 (Figure 6A) and could function in the complete absence of CD4 in the context of high rmCCR5 expression (Figure 1A). It appears that our CD4-independent variants exist constitutively in or spontaneously acquire the CD4-bound conformation, even in the absence of CD4 [9]. Therefore, loss of coreceptor use plasticity may be specific to Envs that have a pre-formed or spontaneously exposed coreceptor binding site [5-8] rather than Envs that scavenge low levels of CD4 on the cell surface [3,4,31-34]. Taken together, these data suggest that diverse mechanisms exist by which virus can expand its host range into CD4-low or negative cells, which may have different consequences for coreceptor interactions. Nevertheless, because these variants arose and came to dominate plasma virus in multiple animals, this mechanism reflects a bona-fide pathway of evolution in this CD4+ T cell depleted model that highlights factors impacting Env function. Whether similar decreased plasticity of coreceptor use is a feature shared by CD4-independent Envs that emerge in other models will be an interesting topic of future studies.

## Conclusion

In conclusion, we have identified loss of coreceptor use plasticity as a potential consequence of CD4-independent entry capacity. The depletion of CD4+ T cell targets in our model likely forced the virus to adapt to a CD4-independent phenotype to enable macrophage targeting, in the permissive environment lacking antibody activity that would otherwise neutralize these variants. An additional consequence of this adaptation, however, is decreased ability to use low levels of CCR5 and related but distinct coreceptor structures. As a result, even though these variants have broadened their range of targets to include CD4-low/CCR5-high cells, they may have a paradoxically narrower range of potential target cells among CD4+ cells that express lower levels or conformational variants of CCR5 such as primary CD4+ T cells. Under normal circumstances, the targeting of such primary CD4+ T cells may be an additional factor that, along with immune pressure, normally limits the emergence of CD4-independent Env variants *in vivo*.

## Methods

### SIV_Mac_ envelope clones and receptor plasmids

The SIV *env* genes analyzed here were cloned by single genome amplification from d42 plasma of SIV-infected rhesus macaques, and mutations introduced using the QuickChange II XL Site-Directed Mutagenesis Kit (Agilent, Santa Clara, CA) and verified by sequencing, as previously described [9,10]. Of 24 Envs cloned from d42 plasma of CD4+ T cell-depleted macaques (animals RUf6, RUt5, RPe6, and RZj5), 23 mediated entry in the absence of CD4 that was >50% of that in the presence of CD4, and were considered “CD4-independent”. None of the 12 Envs cloned from d42 plasma of control macaques (animals RZu4 and KKE) mediated CD4-independent entry, and were designated as CD4-dependent control Envs. Pseudotyped viruses carrying SIV Envs on the NL4.luc/env^-^/vpr^+^ HIV-1 backbone that has luciferase in place of *nef*[36] were generated as previously described [37], and were treated with DNAse prior to use.

### Preparation of target cells

Human 293 T cells were maintained in DMEM containing 10% fetal bovine serum. Coreceptor-transfected target cells were prepared by transfecting 10^6^ 293 T cells with 1 μg plasmid encoding co-receptor with or without 1 μg plasmid encoding CD4, using Fugene transfection reagent (Promega, Madison, WI) as per manufacturer’s instructions, and plasmid pcDNA 3.1 as “filler” to maintain a total of 2 μg plasmid per transfection. To prepare target cells expressing variable levels of CD4 and CCR5, 10^6^ cells were transfected with 1, 0.1, 0.01, or 0 μg plasmid encoding rmCCR5 along with 1, 0.1, or 0 μg plasmid encoding rmCD4, using pcDNA 3.1 as “filler”. One day after transfection, target cells were re-plated into 96-well plates (2x10^4^ per well), and then infected the following day with pseudotyped viruses. In parallel, cells transfected with varying levels of CD4/CCR5 were subject to FACS analysis for CCR5 and CD4 expression using antibodies CD4 (L200)-FITC and CCR5 (3A9)-APC (BD Pharmingen; Franklin Lakes, NJ). For maraviroc titration experiments, 293 T cells were transfected with rmCCR5 and rmCD4 (1 ug each), and treated with 0.1, 1, 10, 100 μM maraviroc (Pfizer, Inc., provided by the NIH AIDS Reagent Repository), or vehicle DMSO alone, for 1 hour prior to and during infection.

Primary human PBMCs from healthy volunteers were cultured at 2x10^6^ cells/mL in RPMI containing 10% fetal bovine serum, stimulated with PHA (5 μg/mL) for 3 days, and maintained thereafter with IL-2 (20 U/mL). On d. 4 after isolation, cells were plated at 10^5^ cells per well in 96-well plates, incubated with or without the CD4 blocking mAb #19 (5 μg/ml; [23]), then infected with pseudotype viruses.

### Infection of target cells

Target cells were infected with Env-pseudotyped viruses (20 ng p24 antigen per virus) by spinoculation at 1200xg for 2 hrs. Cells were then incubated for 72 hrs at 37°C and infection was quantified by measuring luciferase content in cell lysates as previously described [29]. All data represent a minimum of 3 independent experiments, each carried out in duplicate. Where appropriate, entry of each virus into cells expressing the various combinations of CD4 and co-receptor was calculated as a percentage of entry of that virus into cells expressing the highest levels of CD4 and CCR5.

Soluble CD4-183 (sCD4; Pharmacia, Inc.) was obtained from the NIH AIDS Reference Reagents Program, and sCD4 exposure assays were performed essentially as previously described [9]. Pseudotyped virus was mixed with sCD4 to achieve a concentration of virus of 0.8 ng/μl of viral p24 antigen and 50 ng/μl sCD4, and incubated at 37°C for 1 hr. A volume of this mixture (or virus incubated without sCD4) containing 20 ng p24 was used to infect cells as described above.

### Statistical analysis

Statistical analysis was conducted using GraphPad Prism version 4.0c for Macintosh (GraphPad Software, San Diego, CA). Paired T-tests on log-transformed data were used to compare data sets where a single panel of Env variants was tested for entry on 2 different target cell samples, while unpaired T-tests were used for all other mean comparisons. Linear regression analysis was performed using default parameters in Prism, without constraints and fit to mean values. To calculate maraviroc sensitivity, sigmoidal dose response curves and EC50s were generated in Prism, and EC50s compared by Wilcoxon rank-sum test.

## Competing interests

The authors declare that they have no competing interests.

## Authors’ contributions

NF designed and helped carry out all of the experiments, and helped to draft the manuscript. STCE and YY helped design and carry out pseudotype virus entry assays, and STCE performed maraviroc blocking studies. SEG performed mutagenesis and sequence analysis. AMO, BL, MP, GS and CAD generated the SIV envelope clones. RGC, CAD, MP, and GS conceived of the study, participated in its design and coordination, and helped to draft the manuscript. All authors read and approved the final manuscript.
